# Analysis of the correlation between pericoronary adipose tissue mean attenuation and plaque characteristics and stenosis in coronary CT angiography

**DOI:** 10.1097/MD.0000000000037014

**Published:** 2024-02-09

**Authors:** Haolei Liu, Dong Li

**Affiliations:** a Department of Cardiothoracic Imaging, Tianjin Medical University, Tianjin, China; b Department of Cardiothoracic Imaging, Cangzhou People’s Hospital, Cangzhou, China; c Department of Radiology, Tianjin General Hospital of Tianjin Medical University, Tianjin, China.

**Keywords:** atherosclerosis, coronary artery disease, pericoronary adipose tissue mean attenuation, plaque characteristics

## Abstract

Coronary artery disease (CAD) is a predominant cardiovascular disorder, particularly in the aging population. The pathophysiology of atherosclerosis involves lipid deposition and inflammation of the arterial walls. With coronary computed tomography angiography offering insights into coronary anatomy and pathology, parameters such as pericoronary adipose tissue mean attenuation (PCATMA) have gained significance in the understanding of cardiac diseases. A retrospective study encompassing 130 patients with CAD was conducted to analyze 269 observation points. Coronary CT Angiography was employed, with specific attention paid to the measurement of PCATMA and a qualitative and quantitative assessment of plaques. Statistical analyses were performed using Statistical Package for the Social Sciences software (version 27.0), independent samples *t* test, one-way ANOVA, and multivariate logistic regression analysis. There was a notable correlation between PCATMA expression and severity of coronary artery calcification and stenosis. Patients with higher coronary artery calcification scores and more pronounced stenosis had elevated PCATMA values. Variances in PCATMA based on plaque type and degree of stenosis were significant (*P* < .05). Multivariate logistic regression revealed that plaque presence, type, and degree of stenosis were independent determinants of PCATMA expression. PCATMA expression is closely associated with CAD progression. As plaque calcification and arterial stenosis increase, there is a concomitant increase in PCATMA expression, potentially serving as a pivotal prognostic indicator.

## 1. Introduction

Coronary artery disease (CAD) is a major concern in the field of cardiovascular disorders, primarily impacting the older population. Due to the increasingly elderly population in countries like China, there has been a significant increase in the occurrence of CAD.^[1]^ CAD is a major concern in the field of cardiovascular disorders, primarily impacting the older population. Due to the increasingly elderly population in countries like China, there has been a significant increase in the occurrence of CAD.^[2]^ In the development of atherosclerotic lesions, the inflammatory process is crucial and can indicate the likelihood of future cardiovascular events.

Therefore, the inflammation of the coronary arteries has been linked not only to the start and advancement of atherosclerosis, but also shows potential as an early diagnostic indicator for stratifying the risk of CAD. Timely identification and measurement of coronary inflammation can provide valuable information for preventing the development of atherosclerotic plaques, therefore reducing the occurrence of symptomatic CAD.^[3]^ Technological improvements in imaging modalities have provided a noninvasive way to examine the vascular landscape. Coronary computed tomography angiography (CCTA) is now considered a leading technology that offers an exceptional perspective on the structure and abnormalities of the coronary arteries. Parameters obtained from CCTA, particularly the average attenuation of pericoronary adipose tissue mean attenuation (PCATMA), have been shown to be important in predicting outcomes in cardiac diseases.^[4,5]^ PCATMA serves as an imaging biomarker that represents the inflammatory environment around the coronary vessels. It has the potential to reveal the existence and severity of underlying atherosclerotic inflammation.

Nevertheless, the majority of current work has mostly concentrated on the proximal right coronary artery as the designated site for examining the association between PCATMA values and the advancement of CAD and atherosclerosis.^[6,7]^ The limited scope of attention may unintentionally disregard the diversity in the appearance and distribution of atherosclerotic plaques throughout the coronary arteries. Moreover, there is a lack of extensive research that clarifies the connection between PCATMA values and specific attributes of coronary plaques, such as their composition, susceptibility to damage, and the severity of blockage they cause. Given the existing knowledge gaps, our study aims to conduct a comprehensive analysis of the relationship between PCATMA expression and several aspects of plaque features and stenosis in patients with CAD using CCTA. Our objective was to enhance the comprehension of the involvement of coronary inflammation in CAD and provide valuable insights that could drive the development of future diagnostic and therapeutic approaches.

## 2. Materials and methods

### 2.1. Study design

This retrospective study included 130 patients with CAD (68 males and 62 females) who were admitted to Tianjin General Hospital of Tianjin Medical University between May 2021 and May 2023. A total of 269 observation points were evaluated. The age range of the patients was 40 to 73 years, with a mean age of 53.6 ± 5.6 years. The primary objective of this study was to gain a deeper understanding of the clinical profile and outcomes of patients with CAD based on noninvasive imaging. Eligible participants were those aged > 18 years with a definitive diagnosis of CAD ascertained using coronary angiography. Moreover, to maintain sample homogeneity, individuals with a history of interventions, including coronary artery bypass grafting or percutaneous coronary intervention with stent placement, were omitted. To further refine our cohort, we excluded individuals with concurrent endocrine abnormalities, myocarditis, and other potentially confounding cardiac conditions. Additionally, patients presenting contraindications to coronary CT angiography or exhibiting anatomical anomalies in the origin of the coronary vessels were not considered. It is worth noting that our stringent inclusion and exclusion criteria aimed to ensure the validity of our findings and to limit potential confounders. Before enrollment, the significance, objectives, and procedures of the study were explained to all participants and informed consent was obtained. Upholding the highest ethical standards, our research protocol was rigorously reviewed and subsequently sanctioned by the Ethics Committee of the Tianjin General Hospital of Tianjin Medical University.

### 2.2. Coronary CT angiography procedure

The patients underwent coronary CTA scanning using third-generation dual-source CT (Canon Aquilion ONE320-row CT). An initial non-enhanced ECG-gated scan was performed to analyze the coronary artery calcification (CAC) score. The scanning parameters were as follows: tube voltage, 120 kVp; reference tube current, 64 mA; reconstruction slice thickness, 3.0 mm. Subsequently, coronary CTA was performed using CarekV (kVp optimization assistance) with a tube voltage of 70 kVp. ECG-gated high-pitch spiral scans were conducted in patients with a low and steady heart rate after the sublingual administration of nitroglycerin. In cases where patients had heart rates ranging between 70 and 73 beats/min, an oral beta blocker was administered. CTA images were reconstructed with a slice thickness of 0.6 mm. Agatston-based CAC scoring was measured using semi-automatic software (Aquarius iNtuition, TeraRecon, Version 4.4.13), Agatston-based CAC scoring were categorized into 4 classes: 0, 1–99, 100–399, and ≥400.

### 2.3. Measurement of PCATMA

Dedicated software (Aquarius iNtuition, TeraRecon, Version 4.4.13) was used to measure PCATMA in the proximal sections of the right coronary artery (RCA), left anterior descending (LAD), and left circumflex (LCX). Measurements were started 10 mm after the left main bifurcation for the LAD and at the bifurcation point for the LCX, and 10 mm after the RCA orifice. In vessels with plaques, lesion-specific PCATMA measurements were centered on the most significant stenotic plaque. The measurement extended 5 mm proximal and distal to the lesion center, with all measurements having a length and width of 10 and 1 mm, respectively. Considering eccentric plaques, a 1 mm gap between the outer vessel walls was maintained to measure the cylindrical volume, averting artifacts. PCATMA was defined as the mean CT value within the −190 to −30 HU range.

### 2.4. Qualitative and quantitative plaque assessment

Visual assessment was solely employed for plaque presence in the major coronary arteries encompassing the LAD, LCX, and RCA. The most severe plaque in each artery was evaluated for composition and diameter stenosis (DS). Plaques were classified as noncalcified plaques (NCP), mixed plaques, or calcified plaques (CP). In this study, “plaque volume” refers to the total volume of the coronary artery lesion, measured with high precision using VascuCAP software. This volume includes all components of the plaque, such as calcified, non-calcified, and mixed components. Based on plaque density and volume, the software automatically categorizes them into calcified, non-calcified, and mixed plaques. Using visual analysis, CP was defined when the plaque volume was >75% with a density surpassing the lumen contrast, whereas NCP was defined when the plaque volume was >75% with a density lower than the lumen contrast yet higher than the surrounding soft tissues. Mixed plaques contained 25% to 75% volume, with a density exceeding the lumen contrast. DS was categorized into 4 groups: minimal (DS 1–24%), mild (DS 25–49%), moderate (DS 50–69%), and severe (DS 70–100%).

For quantitative plaque composition, semi-automated software (VascuCAP, research version by Elucid Bioimaging) was used. Alongside visual analysis, VascuCAP software’s quantitative functionalities were utilized to assist in plaque component assessment. This software accurately identifies and quantifies the calcified, non-calcified, and mixed components of the plaque. The combination of visual analysis with quantitative data from the software allows for a more precise and objective assessment of plaque type and composition. Automatic segmentation of the entire coronary lumen and vessel wall was performed with manual correction permitted when necessary. The matrix, CP, and lipid-rich necrotic core (LRNC) loads were computed automatically on a per-vessel basis. Histologically validated classifications for various plaque types were based on the adaptive threshold values. The lower limit for LRNC was defined as −300 HU, while the boundary for LRNC internal plaque hemorrhage (LRNC-IPH) was set at −25 HU. CP had lower and upper limits of 250 HU and 3000 HU, respectively. Matrix load calculation entailed dividing the total wall volume by the matrix volume, with the matrix defined as normal tissue within the vessel wall. Plaque load was defined as 1 minus the matrix load.

### 2.5. Statistical analysis

Statistical analyses were performed using IBM’s Statistical Package for the Social Sciences software, version 27.0. Quantitative data are presented as mean ± standard deviation, providing a clear depiction of data distribution and variability. To ascertain differences between 2 distinct groups, an independent sample *t* test was performed. In situations with multiple group comparisons, a one-way ANOVA test was preferred to ensure accurate discernment of group variances. Furthermore, to delve deeper into potential predictors and associations within the dataset, a multivariate logistic regression analysis was conducted, allowing us to control for confounding variables and determine the strength and direction of the associations between predictors and outcomes. Throughout the analysis, a stringent *P* value threshold of less than .05 was adhered to, ensuring that the findings were statistically significant and not merely the result of random chance.

## 3. Results

### 3.1. Correlation of PCATMA with coronary artery calcification and stenosis severity

The intricate relationship between PCATMA expression, CAC, and stenosis severity has been a subject of increasing interest. As shown in Table 1, a significant and progressive increase in PCATMA expression was observed with increasing CAC scores and enhanced stenosis severity. Specifically, patients with higher CAC scores and more pronounced stenosis had elevated PCATMA values. The data from the present study underscore the potential of PCATMA as a predictive marker, helping clinicians to understand the degree of arterial calcification and stenosis in patients. These findings emphasize the clinical implications of these parameters for the prognostic evaluation of cardiovascular conditions. Statistical assessments confirmed these observations and revealed significant differences (*P* < .05).

**Table 1:** PCATMA expression relative to distinct CAC scores and stenosis degrees.

Parameters	n	PCATMA (HU ± SD)	*F* value	*P* value
CAC score				
0	41	−92.10 ± 7.10	4.78	0.004
1–89	34	−95.40 ± 7.20		
90–360	31	−96.50 ± 7.30		
≥360	24	−97.00 ± 7.50		
DS				
1–24%	33	−93.80 ± 7.40	3.40	0.029
25–49%	56	−95.20 ± 7.10		
50–69%	31	−96.40 ± 7.90		
70–100%	10	−97.40 ± 7.50		

CAC = Coronary Artery Calcification, DS = Diameter Stenosis, HU = Hounsfield Units, PCATMA = pericoronary adipose tissue mean attenuation, SD = standard deviation.

### 3.2. PCATMA expression variances in relation to plaque nature and stenosis degree

This study assessed the differences in PCATMA expression contingent on plaque characterization and degree of stenosis, both in proximal and lesion-specific zones. As portrayed in Table 2, variations in PCATMA expression across plaque types were statistically significant (*P* < .05), with lesion areas showing slightly augmented PCATMA expression relative to their proximal counterparts. Nevertheless, the comparative deviation between PCATMA values of the proximal and lesion areas remained statistically non-significant (*P* > .05), indicating that while the lesion zones might reflect a marginally higher PCATMA expression, the distinction is not prominent enough to be deemed statistically relevant (Table 3).

**Table 2:** Differences in PCATMA expression based on plaque types.

Plaque types	Non-calcified (n = 34)	Mixed (n = 46)	Calcified (n = 50)	*F* value	*P* value
Proximal PCATMA	−95.50 ± 7.00	−97.20 ± 7.10	−98.80 ± 7.40	4.200	0.011
Lesion PCATMA	−96.30 ± 7.20	−97.80 ± 7.30	−99.60 ± 7.60	3.800	0.022
*t*	0.770	0.520	0.550	–	–
*P*	0.460	0.510	0.550	–	–

PCATMA = pericoronary adipose tissue mean attenuation.

**Table 3:** Differences in PCATMA expression based on stenosis degree.

Stenosis degree	1–24% (n = 33)	25–49% (n = 56)	50–69% (n = 31)	70–100% (n = 10)	*F* value	*P* value
Proximal PCATMA	−94.40 ± 7.60	−95.90 ± 8.00	−97.50 ± 8.10	−99.10 ± 8.40	3.200	0.033
Lesion PCATMA	−95.10 ± 7.80	−96.60 ± 8.20	−98.20 ± 8.30	−99.80 ± 8.50	2.900	0.030
*t*	0.800	0.880	0.790	0.450	–	–
*P*	0.450	0.380	0.420	0.650	–	–

PCATMA = pericoronary adipose tissue mean attenuation.

### 3.3. PCATMA expression and its association with plaque characteristics and stenosis

To better understand the underlying factors influencing PCATMA expression, multivariate logistic regression analysis was conducted. The findings summarized in Table 4 emphasize that the presence of a plaque, its specific type, and the degree of stenosis all serve as independent determinants affecting the expression of PCATMA (*P* < .05). Notably, while each of these factors individually bore a significant relationship with PCATMA expression, their combined effects shed light on the intricate dynamics between coronary plaque attributes and pericoronary adipose tissue characteristics.

**Table 4:** Multivariate logistic regression analysis of PCATMA expression in relation to plaque presence, plaque type, and stenosis degree.

Variables	β	Odds ratio	95% confidence interval	*P* value
Plaque presence	0.805	2.300	−98.50 to −93.90	.015
Plaque types				
Non-calcified	1.320	3.650	−99.10 to −92.50	<.001
Mixed	1.505	3.900	−96.90 to −91.50	<.001
Calcified	0.880	2.400	−96.50 to −89.70	.002
Stenosis degree				
1–24%	0.712	2.030	−97.50 to −92.30	.082
25–49%	1.330	3.780	−97.10 to −89.20	.003
50–69%	0.860	2.360	−97.80 to −87.70	.007
70–100%	0.810	2.250	−101.30 to −93.70	.028

PCATMA = pericoronary adipose tissue mean attenuation.

## 4. Discussion

The complexities of CAD go beyond simple stenoses or anatomical alterations. CAD, typically shown as the constriction or obstruction of blood channels in the heart, is not solely a mechanical issue. Instead, it involves an intricate interplay of multiple pathophysiological mechanisms. This complex ailment emerges from a blend of inherent genetic tendencies, disrupted metabolic processes, impaired functioning of blood vessel lining, and inflammatory reactions. The interaction between these components is dynamic, resulting in the creation, progression, and possible rupture of plaque.^[8]^ The analysis of the makeup of atherosclerotic plaques and the extent of stenosis provides important information about the susceptibility of the plaques and their impact on blood flow dynamics. Plaques that are susceptible to rupture, typically identified by a thin fibrous cap and a sizable lipid-rich necrotic core, have a tendency to cause sudden coronary events. The degree of stenosis, however, directly influences the extent to which blood flow is restricted in the afflicted blood channel. The type of plaque and the severity of stenosis have a crucial role in guiding therapeutic therapies and predicting the likelihood of future cardiac events.^[9,10]^ We investigate the correlation between PCATMA and the presence, kind, and intensity of coronary artery stenosis in symptomatic patients who are having CCTA at 70 kVp.

Convincing evidence indicates a substantial rise in PCATMA expression as both CAC score and stenosis level increase. Different types of plaques and different levels of narrowing in the arteries also show noticeable differences in the expression of PCATMA in the proximal area and in the specific lesion. The expression in the lesion is slightly higher compared to the proximal area, albeit not significantly. Importantly, the absence of notable distinction between the 2 regions corresponds with our comprehensive statistical study using multivariate logistic regression. This analysis identified plaque presence, type, burden, and stenosis degree as separate factors that influence the expression of PCATMA. Prior studies indicated a discrepancy of around 3 HU in the PCATMA based on the RCA between patients with CAD and those without CAD, with CAD being defined as having more than 50% narrowing of the artery. The PCATMA values of a person are influenced by the distribution of coronary artery and plaque, with the LAD artery often exhibiting variability. Relying exclusively on the RCA as a benchmark for PCATMA may not yield a precise depiction of the patient’s PCATMA condition. Oikonomou et al^[11]^ reported a connection between increased PCATMA in the RCA and LAD and increased cardiac mortality risk. Gaibazzi et al,^[12]^ on the other hand, detailed a remarkable difference in the LAD/RCA and LCX in vessels with stenosis less than 50%, wherein HU discrepancy was approximately 1.5 HU at 120 kVp scanning.

Beyond the coronary arteries, the measurement location can profoundly influence PCATMA. Although a correlation exists between PCATMA and Epicardial Adipose Tissue, no discernible link exists between Epicardial Adipose Tissue changes and plaque burden progression. Dai et al^[13]^ did not find any connection between lesion-specific PCATMA and high-sensitivity C-reactive protein, suggesting localized coronary artery inflammation for PCATMA as opposed to systemic inflammation.^[13,14]^ Few studies have explored the relationship with coronary artery plaques; however, Kwiecinski et al noted that an increase in lesion-specific PCATMA was related to focal ^18^F-NaF PET uptake in high-risk plaque patients.^[15]^ Lin et al^[16]^ examined the relationship between radiomics features and PCATMA in the proximal RCA and its surroundings during initial visits and 6 months post-treatment in stable CAD and non-CAD cases. Radiomics parameters, such as texture and geometric shape, have emerged as pivotal discriminators between patients with and without plaques, thereby offering insights not encapsulated within PCATMA attenuation. However, they discovered significant differences in lesion-specific PCATMA expression, and proposed PCATMA as a potential lesion-specific imaging biomarker. Acknowledging factors such as age, sex, coronary arteries, and lesion specific PCATMA vary with DS categories.

Our findings emphasize that mild and moderate DS may be more inflamed than severe DS. Two potential mechanisms can be hypothesized: inflammation may decrease as plaques become more stable and calcified in severe DS; and cytokines play a pivotal role in the development and progression of atherosclerosis, with the theory behind PCATMA being that atherosclerosis in the vascular wall inhibits adipocyte maturation and lipid accumulation in pericoronary adipose tissue, leading to increased attenuation.^[17]^ The link between coronary inflammation and PCATMA may be more evident in NCP than in CP, since CP is relatively stable with minimal inflammatory components. Recent studies have shown that LRNC burden predicts myocardial infarction better than CAC scores, cardiovascular risk scores, and coronary artery stenosis.

## 5. Conclusions

PCATMA expression is intimately linked to the onset and progression of CAD. As patients exhibit increased plaque calcification and arterial stenosis, PCATMA expression shows an upward trend. This may serve as a pivotal indicator of patient prognosis.

## Author contributions

**Data curation:** Haolei Liu.

**Formal analysis:** Haolei Liu.

**Investigation:** Haolei Liu.

**Methodology:** Haolei Liu.

**Resources:** Haolei Liu.

**Supervision:** Dong Li.

**Visualization:** Dong Li.

**Writing – original draft:** Haolei Liu.

**Writing – review & editing:** Dong Li.

## References

[1] Malakar AK Choudhury D Halder B. A review on coronary artery disease, its risk factors, and therapeutics. J Cell Physiol. 2019;234:16812–23. 30790284 10.1002/jcp.28350

[2] Libby P Theroux P. Pathophysiology of coronary artery disease. Circulation. 2005;111:3481–8. 15983262 10.1161/CIRCULATIONAHA.105.537878

[3] Hansson GK. Inflammation, atherosclerosis, and coronary artery disease. N Engl J Med. 2005;352:1685–95. 15843671 10.1056/NEJMra043430

[4] Antonopoulos AS Sanna F Sabharwal N. Detecting human coronary inflammation by imaging perivascular fat. Sci Transl Med. 2017;9:eaal2658. 28701474 10.1126/scitranslmed.aal2658

[5] Oikonomou EK Williams MC Kotanidis CP. A novel machine learning-derived radiotranscriptomic signature of perivascular fat improves cardiac risk prediction using coronary CT angiography. Eur Heart J. 2019;40:3529–43. 31504423 10.1093/eurheartj/ehz592 PMC6855141

[6] Elnabawi YA Oikonomou EK Dey AK. Association of biologic therapy with coronary inflammation in patients with psoriasis as assessed by perivascular fat attenuation index. JAMA Cardiol. 2019;4:885–91. 31365032 10.1001/jamacardio.2019.2589 PMC6669789

[7] Elnabawi YA Dey AK Goyal A. Coronary artery plaque characteristics and treatment with biologic therapy in severe psoriasis: results from a prospective observational study. Cardiovasc Res. 2019;115:721–8. 30721933 10.1093/cvr/cvz009 PMC6432047

[8] Sutton NR Banerjee S Cooper MM. Coronary artery disease evaluation and management considerations for high risk occupations: commercial vehicle drivers and pilots. Circ Cardiovasc Interv. 2021;14:e009950. 34092098 10.1161/CIRCINTERVENTIONS.120.009950

[9] Stone PH Libby P Boden WE. Fundamental pathobiology of coronary atherosclerosis and clinical implications for chronic ischemic heart disease management-the plaque hypothesis: a narrative review. JAMA Cardiol. 2023;8:192–201. 36515941 10.1001/jamacardio.2022.3926 PMC11016334

[10] Severino P D’Amato A Pucci M. Ischemic heart disease pathophysiology paradigms overview: from plaque activation to microvascular dysfunction. Int J Mol Sci. 2020;21:8118. 33143256 10.3390/ijms21218118 PMC7663258

[11] Oikonomou EK Marwan M Desai MY. Non-invasive detection of coronary inflammation using computed tomography and prediction of residual cardiovascular risk (the CRISP CT study): a post-hoc analysis of prospective outcome data. Lancet. 2018;392:929–39. 30170852 10.1016/S0140-6736(18)31114-0 PMC6137540

[12] Gaibazzi N Martini C Botti A. Coronary inflammation by computed tomography pericoronary fat attenuation in MINOCA and Tako-Tsubo syndrome. J Am Heart Assoc. 2019;8:e013235. 31462127 10.1161/JAHA.119.013235 PMC6755824

[13] Dai X Deng J Yu M. Perivascular fat attenuation index and high-risk plaque features evaluated by coronary CT angiography: relationship with serum inflammatory marker level. Int J Cardiovasc Imaging. 2020;36:723–30. 31907683 10.1007/s10554-019-01758-8

[14] Yu M Dai X Deng J. Diagnostic performance of perivascular fat attenuation index to predict hemodynamic significance of coronary stenosis: a preliminary coronary computed tomography angiography study. Eur Radiol. 2020;30:673–81. 31444596 10.1007/s00330-019-06400-8

[15] Kwiecinski J Dey D Cadet S. Peri-coronary adipose tissue density is associated with (18)F-sodium fluoride coronary uptake in stable patients with high-risk plaques. JACC Cardiovasc Imaging. 2019;12:2000–10. 30772226 10.1016/j.jcmg.2018.11.032 PMC6689460

[16] Lin A Kolossváry M Yuvaraj J. Myocardial infarction associates with a distinct pericoronary adipose tissue radiomic phenotype: a prospective case-control study. JACC Cardiovasc Imaging. 2020;13:2371–83. 32861654 10.1016/j.jcmg.2020.06.033 PMC7996075

[17] Tousoulis D Economou EK Oikonomou E. The role and predictive value of cytokines in atherosclerosis and coronary artery disease. Curr Med Chem. 2015;22:2636–50. 25876746 10.2174/0929867322666150415145814

